# Mental disorders among adults formerly in out-of-home care: a systematic review and meta-analysis of longitudinal studies

**DOI:** 10.1007/s00787-021-01828-0

**Published:** 2021-06-24

**Authors:** Süheyla Seker, Cyril Boonmann, Heike Gerger, Lena Jäggi, Delfine d’Huart, Klaus Schmeck, Marc Schmid

**Affiliations:** 1 Department of Child and Adolescent Psychiatry Research, Psychiatric University Hospitals, University of Basel, Wilhelm Klein-Strasse 27, 4002 Basel, Switzerland; 2 Division of Clinical Psychology and Psychotherapy, Faculty of Psychology, University of Basel, Basel, Switzerland; 3 Department of General Practice, Erasmus MC University Medical Center, Rotterdam, The Netherlands; 4 Division of Personality and Developmental Psychology, Faculty of Psychology, University of Basel, Basel, Switzerland

**Keywords:** Mental disorder, Developmental psychopathology, Child welfare system, Juvenile justice system, Systematic review, Meta-analysis

## Abstract

While children and adolescents placed in child welfare or juvenile justice out-of-home care show higher prevalence rates of mental disorders compared to the general population, it remains unclear whether this pattern persists into adulthood. A quantitative synthesis of existing studies is lacking. The aim of this meta-analysis was to estimate the prevalence rates for mental disorders among adults with a foster or residential child welfare or juvenile justice care history, comparing them where possible to rates among the general population. PubMed, PsycInfo, EMBASE, and Web of Science were systematically searched for epidemiological studies published up to 28 October 2020. Nineteen studies, totaling 604,257 participants, met our inclusion criteria. Random-effects models were used for prevalence rates and odds ratios (OR) of mental disorders, and study quality was rated. A prevalence rate of 30% [95% CI (23.36, 37.36)] for any mental disorder in adults with a child welfare care history was found (3–17% for specific disorders). A prevalence rate of 45% [95% CI (42.38, 47.38)] for any mental disorder was found in adults with a juvenile justice care history (6–66% for specific disorders). For out-of-home placement history, adult mental disorders were significantly higher than in the general population (OR = 1.33–2.76). Studies differed in terms of methodology and the disorder groups considered, so heterogeneity between effect sizes ranged from low to high. Our findings suggest that the high risk that mental health issues will persist in adults with an out-of-home placement history needs to be taken seriously in the transition from adolescence to adulthood. The care systems involved need to collaborate and to be aware of these risks.

**Supplementary Information:**

The online version contains supplementary material available at 10.1007/s00787-021-01828-0.

## Introduction

Millions of children and adolescents worldwide are placed in out-of-home care, with rates ranging from approximately 0.5–1% between countries [1, 2]. The prevalence of mental disorders is high in these children and adolescents [3, 4], as they face various challenges related to mental disorders in the transition period from leaving care to adulthood (e.g., homelessness, adult mental disorders, or unemployment; [5–7]). To date, the extent of the association between placement in childhood out-of-home care and the occurrence of adult mental disorders remains unclear. Thus, the aim of our systematic review and meta-analysis is to provide an overview of prevalence rates for mental disorders (specific and overall) among adults previously in child welfare or juvenile justice out-of-home care. These findings will inform researchers and professionals alike about the burden of mental health diagnoses in this highly vulnerable population.

Children and adolescents in out-of-home care can generally be placed there either by child welfare (civil law) or juvenile justice (criminal law) authorities. The quality of structures and processes in different care settings within and across child welfare and juvenile justice systems varies substantially between countries with different socioeconomic opportunities and policies [8–11]. In general, adolescent offenders are marked by highly elevated rates of trauma, psychopathology, and other psychosocial problems [12, 13], while adolescents in child welfare placements often show delinquent behavior in addition to similarly elevated rates of psychosocial treatment needs [3, 14]. A substantial number of child welfare adolescents thus cross over to the juvenile justice system or vice versa, meaning that these adolescents can be involved in both care systems [15, 16]. These crossover adolescents show higher rates of psychopathology, greater placement instability, and penetrate deeper into both systems, making it particularly important for professionals to reach across systems of care to support at-risk individuals [17]. Although children and adolescents involved with child welfare or juvenile justice authorities overlap and experience similarly high levels of psychosocial burden and childhood adversities [15], combining child welfare and juvenile justice samples may obscure significant differences in the prevalence rates of mental disorders. Therefore, for child welfare placement histories, we distinguish between foster care and residential care; and for residential care histories, we distinguish between child welfare placements and juvenile justice placements.

A previous meta-analysis of studies conducted in Europe and the United States showed that 49% (including present and lifetime diagnoses) of children and adolescents in the child welfare system (including residential and foster care settings) met the criteria for at least one present mental disorder [3]. These rates ranged from 27% for disruptive disorders to 4% for post-traumatic stress disorders [3]. Furthermore, prevalence rates can vary between different care settings [18, 19]. For example, a rate of 74% for any lifetime mental disorder was found in a study examining adolescents in Swiss residential care settings [20], compared with the 51% for any lifetime mental disorder reported in a sample of foster adolescents in Norway [21]. Among juvenile justice samples, a prevalence rate of 70% for any mental disorder (including present and lifetime diagnoses) was found, with rates ranging from 45% for conduct disorders to 1% for psychotic disorders (see the literature review of Colins et al. [22]). A recent meta-analytic study of adolescents in juvenile detention and correctional facilities reported high present and lifetime prevalence rates, ranging from 3% for psychotic disorders to 63% for conduct disorders [4]. In comparison, a meta-analysis examining the general adolescent population internationally estimated a combined present and lifetime prevalence rate of 13% for any mental disorder [3, 4, 23]. In addition, previous meta-analyses also showed that a stable foster or residential care placement can lower psychosocial burden and be protective against high levels of psychopathology among high-risk children [24, 25]. Furthermore, foster care showed slightly better outcomes for behavioral problems and social and cognitive skills compared to institutionalization (i.e., group care), and no differences between care settings were found when the institutional treatments were evidence-based [26]. While epidemiological data for childhood and adolescence allow robust conclusions, the long-term perspective remains unclear, and an up-to-date meta-analytic summary of epidemiological studies investigating the occurrence of mental disorders in adults with a history of out-of-home placement is lacking.

From a developmental perspective, monitoring the mental state of children and adolescents in child welfare or juvenile justice care placements is crucial, as disorders that manifest in childhood or early in life carry a high risk of persisting into adulthood and severely influencing long-term quality of life and functionality [27, 28]. Young adulthood in particular can be a vulnerable period where the development and chronicity of mental disorders are concerned [29, 30]. Yet to date, only a few studies of representative general population samples have examined the course of mental disorders from childhood to adulthood. Overall, these studies reported high levels of persistence of mental disorders into adulthood, especially for individuals with numerous risk factors [27, 31–34]. Similarly, prospective and retrospective studies have emphasized that adolescents who were placed within the juvenile justice system suffer from multiple mental health problems in young adulthood [35–38]. Furthermore, primary studies have shown that the challenging transition from foster or residential care to an independent adult life is associated with poor mental health for such emerging adults [7, 39, 40]. Longitudinal studies with transition-aged young adults from care have focused on various outcomes of psychosocial functioning and general mental health [39, 41], and a literature review has shown that the level of psychopathology among young adults who aged out of the child welfare system was high overall [42].

In summary, previous meta-analyses have examined the prevalence or development of mental health disorders from childhood through adolescence among individuals placed in care by either the child welfare [25, 41, 43] or the juvenile justice system [4, 22]. However, to the best of our knowledge, no systematic review or meta-analysis has investigated the burden of mental disorder among the high-risk sample of adults with a history of out-of-home care. The aim of our systematic review with a meta-analysis is to provide a comprehensive overview of the prevalence rates of mental disorders among adults formerly in out-of-home care in existing studies, distinguishing between child welfare foster or residential care on the one hand and juvenile justice residential care settings on the other. Furthermore, where possible, the rates of mental disorders will be compared with the occurrence of mental disorders in available control groups. An overview of mental disorders among adults formerly in out-of-home care has the potential to inform the development of targeted preventive interventions for adult mental disorders in individuals with a history in different out-of-home care settings.

## Methods

The present systematic review and meta-analysis were conducted in accordance with the Meta-Analyses of Observational Studies in Epidemiology (MOOSE) guidelines (see Supplementary Content 1; [44]). The review protocol was registered in PROSPERO (International Prospective Register of Systematic Reviews; registration number: CRD42019141330); deviations of the final review from the initial protocol are explained in Supplementary Content 2. The advice of a research librarian was obtained for the literature search.

### Search strategy and study selection

The literature searches were conducted in the electronic databases PubMed, PsycInfo, EMBASE, and Web of Science on 15 November 2019 and updated on 28 October 2020. We used keywords and Medical Subject Headings (MeSH) terms to identify peer-reviewed journal articles reporting prevalence rates of mental disorders among adults with a foster or residential care history, including articles on juvenile justice placements. We did not set any time limitation for published articles. The search terms used in the individual databases are presented in Supplementary Content 3.

Our inclusion criteria for articles were that they be English-language, peer-reviewed case–control or cohort studies (both prospective and retrospective), reporting prevalence rates for mental disorders in adults with a history of foster care or residential care in childhood or adolescence. If multiple time periods for prevalence rates of diagnoses were reported, we chose the present or 1-year prevalence rates; lifetime prevalence rates were only included if they were the only rates reported and subgroup analyses allowed childhood prevalences to be excluded. We excluded reports, comments, letters, gray literature, intervention studies, and reviews, as well as qualitative studies.

The screening and selection processes were conducted independently by two authors (SS and LJ) using Covidence, an online screening and data extraction tool based on Cochrane reviews (https://www.covidence.org/home).

### Outcome measures and data extraction

Our primary outcome was the presence or absence of mental disorders. Mental disorders were defined following either the International Classification of Diseases (ICD) or *Diagnostic and Statistical Manual of Mental Disorders* (DSM). Mental disorders were assessed by means of hospital reports, (semi-)structured interviews (e.g., Composite International Diagnostic Interview [CIDI]), and well-validated screening scales (e.g., the General Health Questionnaire [GHQ] and the Malaise Inventory; [45, 46]). Registry data from official hospital reports or surveys of mental disorder diagnoses were also included. For the calculation of prevalence rates, we extracted the number of cases (i.e., study participants with mental disorders) and non-cases (i.e., study participants without a mental disorder) from all studies identified by the systematic review. For the calculation of odds ratios (OR), we used the subset of studies that included control groups. We extracted the 2 × 2 cross-tabulation data for number of cases and non-cases for the control group as well (i.e., adults without an out-of-home care history).

Data were extracted independently by two authors (SS and DdH). The discrepancies in author coding assignments were discussed and resolved by consensus. Extracted data included demographic information about the sample (i.e., sample size, gender, and age), type of care setting in child welfare (i.e., foster care, kinship care, residential care, or adoption) and juvenile justice (i.e., juvenile detention facility or correctional facility) systems, age at entry into care (in years), duration of care (in months or years), location (i.e., United States, Europe, Oceania, Africa, or Asia), study design (i.e., prospective or retrospective), dropout rates (in %), outcome measures (i.e., the diagnostic instrument), time period of prevalence (i.e., present, 1-year, or lifetime), and outcomes (i.e., mental disorder groups). We included five categorical variables (i.e., type of care setting in child welfare and juvenile justice contexts, location, study design, outcome measure, and time period of prevalence) as potential moderators in our subgroup analyses. In the case of studies with more than one follow-up assessment, we extracted data for the last available assessment in adulthood. The data extracted from six studies were incomplete; after repeated attempts to obtain the full data, we received them from the two authors [47, 48] and were able to include their two studies in our study. We were unable to obtain full data from the other four studies [49–52], which were, therefore, excluded from our study.

### Methodological quality assessment

Two authors (SS and DdH) independently rated the methodological quality of the included studies on the basis of the Newcastle–Ottawa Scale (NOS; [53]). This tool was developed for the quality assessment of non-randomized studies and evaluates them in three broad domains: selection of the study groups (rated with the “Representativeness of the Exposed Cohort”, “Selection of the Non-Exposed Cohort”, “Ascertainment of Exposure”, and “Demonstration that Outcome of Interest Was Not Present at Start of Study” criteria), comparability of the cohort groups (rated with the “Comparability of Cohorts on the Basis of the Design or Analysis” criterion), and ascertainment of the exposure or outcome of interest for case–control or control studies, respectively (rated with the “Assessment of Outcome”, “Was Follow-Up Long Enough for Outcomes to Occur”, and “Adequacy of Follow-Up of Cohorts” criteria). To rate the comparability of study cohort groups, the NOS requires one or two key control variables to be predetermined. We included age and/or gender as the first control variable(s), and information on socioeconomic status as a further control variable. A previous study has shown higher rates of boys in out-of-home care than girls, different rates of mental disorders depending on age, and that out-of-home placed individuals originated from families with lower socioeconomic backgrounds compared to the general population [54]. If two of the predefined control variables were met, the “Comparability of Cohorts on the Basis of the Design or Analysis” criterion was scored with 2 points.

Each study was rated in terms of eight criteria (seven criteria were scored 0 or 1, and one criterion was scored 0, 1, or 2), resulting in a maximum score of 9 for each study. The final quality ratings were based on consensus between the two authors (SS and DdH) after discrepancies in the coding assignments were discussed and resolved. The quality of a study was assessed as *high* when seven to nine criteria were met, *moderate* when four to six criteria were met, and *low* when three or fewer criteria were met.

### Statistical analyses

First, the prevalence rates of mental disorders (both any and specific disorders) were calculated by dividing the number of adults with a mental disorder by the full sample size of adults with an out-of-home placement history. Second, ORs for the individual disorders were calculated based on the percentage of all individuals with a mental disorder among the total sample and among those with foster or residential care histories (child welfare or juvenile justice placements) as well as control groups.

For the meta-analytic aggregation of effect sizes, we chose random-effects models rather than a fixed-effect model because we assumed significant variability in clinical assessment and methodology between the studies included [55]. Between-study heterogeneity of results was assessed by calculating the *Q* statistic, *τ*^2^, and *I*^2^*.* *τ*^2^ offers an estimate of the variance between true effect sizes and is not sensitive to sample size, in contrast to *I*^2^ [56, 57]. *I*^2^ is a transformation of *Q* that indicates the proportion of observed variance that can be attributed to heterogeneity rather than to sampling error [58]. In general, this number can be interpreted as follows: a percentage of 25% is assumed to indicate low, 50% moderate, and 75% high heterogeneity [59].

Subgroup analyses were conducted for five categorical moderators (i.e., type of care setting in child welfare and juvenile justice contexts, location, study design, outcome measure, and time period of prevalence) for the specific disorder groups. Subgroup analyses for individual disorders were conducted if more than 10 studies could be included in them [55]. Sensitivity analyses to reduce heterogeneity were conducted for prevalence rates and ORs by excluding outlier studies, defined as studies falling outside the total confidence interval range of the respective disorder group. Publication bias was assessed visually by means of funnel plots. The “meta” package in R was used for all analyses and plots (version 4.0.2; [60]). We used a 2-sided *p* < .05 to indicate statistical significance.

## Results

### Characteristics of included studies

**Fig. 1:**
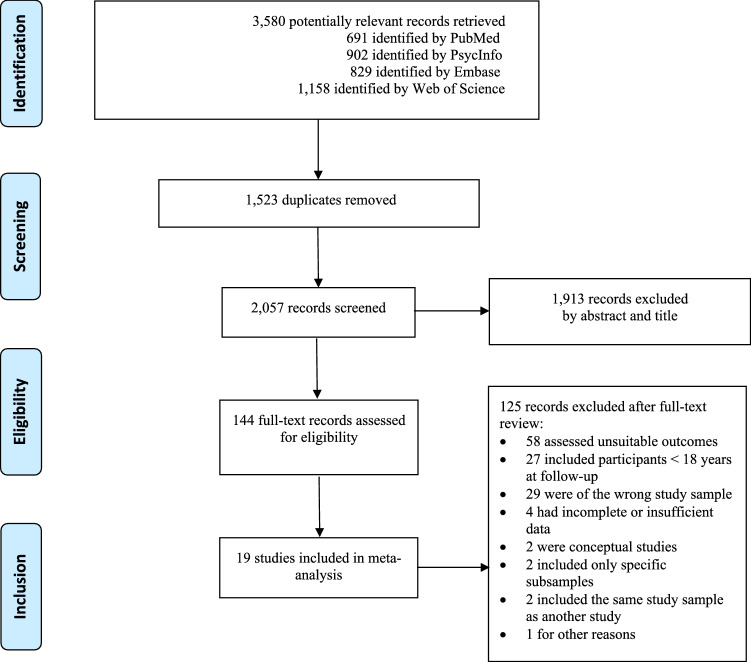
Flowchart of study inclusion

In total, the systematic search of electronic databases revealed 3,580 potentially relevant articles (see Fig. 1 for the flowchart of study inclusion). The screening and full-text assessment resulted in 19 peer-reviewed journal articles with case–control and cohort studies, with a total sample of 604,257 participants. For a complete list and general study characteristics, see Table 1.

**Table 1:** Characteristics of included studies (*k* = 19)

Study	Sample characteristics^a^	Setting(s)	Age at entry	Duration of care	Location	Study design	% Dropout	Outcome measure(s)^b^	Time period of prevalence	Outcome(s)
Child welfare placement history										
Benedict et al. [70]	*N* = 214 55% female Mean age = 23 years	Foster care, kinship care	38% < 5 years	Median = 12 years	United States	Prospective	31.2	GHQ	Present	Any mental disorder
Brown et al. [76]	*N* = 602 53.5% female Mean age = 23.91 years	Foster care	N/R	N/R	United States	Prospective	18	CIDI	1 year	Any mental disorder, depressive disorders, post-traumatic stress disorders, substance-use disorders
Forsman et al. [47]	*N* = 7,522 47.55% female Age range = 30–35 years	Foster care	N/R	Mean = 4.99 years (SD = 5.79)	Europe	Prospective	N/R	Hospital records	Present	Any mental disorder
Hjern et al. [103]	*N* = 371 48.85% female Age range = 30–39 years	Foster care, adoption	Mean age = 2.6 years	Mean = 8.4 years	Europe	Prospective	N/R	Hospital records	Present	Any mental disorder, substance-use disorders
Landers et al. [48]	*N* = 233 81% female Mean age = 48.96	Foster care	N/R	N/R	United States	Retrospective	N/A	Survey	Present	Any mental disorder, depressive disorders, eating disorders, substance-use disorders
Räikkönen et al. [68]	*N* = 12,747 47.4% female Median age = 41.3 years	Foster care, CG: general population sample	Mean age = 4.6 years (SD = 2), age range = 0.17–10.6 years	Mean = 1.7 years (SD = 1.6), range = 0.05–8.1 years	Europe	Prospective	1.95	ICD-8, ICD-9, ICD-10	Present and lifetime	Any mental disorder, substance-use disorders, psychotic disorders, depressive disorders, anxiety disorders
Björkenstam et al. [65]	*N* = 478,141 49% female Age range = 22–24 years	Mixed samples in out-of-home care, CG: general population sample	< 12 years	N/R	Europe	Prospective	N/R	ICD-10	Present	Depressive disorders
Casanueva et al. [77]	*N* = 802 58.5% female Age range = 18–21 years	Mixed samples in out-of-home care	N/R	Mean = 15 years	United States	Prospective	16.8	CIDI-SF	1 year	Substance-use disorders
Côté et al. [67]	*N* = 54,814 Mean age = 49 years Age range = 18–25 years	Mixed samples in out-of-home care, CG: general population sample	Age range = 2–6 years	N/R	Europe	Prospective	7.8	ICD-10, ICD-9	Present	Any mental disorder, substance-use disorders
Dregan and Gulliford [66]	*N* = 10,895 51% female Mean age = 30 years	Mixed samples in out-of-home care, foster care, residential care, CG: general population sample	Age range = 5–30 years	Residential care = 1–3 months, foster care = 4–12 months, residential and foster care ≥ 1 year	Europe	Prospective	42	The Malaise Inventory, CAGE Scale	1-year	Depressive disorders, substance-use disorders
Fernandez et al. [78]	*N* = 67 22.7% female Mean age = 71.5 years	Mixed samples in out-of-home care	N/R	N/R	Oceania	Retrospective	N/A	Survey	Present	Any mental disorder
Southerland et al. [69]	*N* = 620 59.7% female Age range = 18–21 years	Mixed samples in out-of-home care, CG: general population sample	N/R	N/R	United States	Prospective	14.7	CIDI-SF	Present	Any mental disorder
Teyhan et al. [61]	*N* = 12,429 70% female Mean age = 30.5 years	Mixed samples in out-of-home care and adoption, CG: general population sample	≤ 18 years	N/R	Europe	Retrospective	5.6	EPDS, CCEI	Present	Anxiety disorders, depressive disorders
Viner and Taylor [62]	*N* = 9,557 51.9% female Mean age = 30 years	Mixed samples in out-of-home care, CG: general population sample	< 17 years	N/R	Europe	Prospective	42.3	The Malaise Inventory, CAGE Scale	Present	Any mental disorder, substance-use disorders
Vinnerljung and Sallnäs [63]	*N* = 698 50.6% female Age range = 20–24 years	Mixed samples in out-of-home care, CG: general population sample	Age range = 13–16 years	N/R	Europe	Prospective	8	ICD	Present	Any mental disorder
Juvenile justice placement history										
Abram et al. [71]	*N* = 1,520 38% female Mean age = 27.6 years	Juvenile detention facilities	Age range = 10–12 years or 14–18 years	N/R	United States	Prospective	17	DISC-IV, DIS-IV, WMH-CIDI	1 year	Any mental disorder, substance-use disorders
Heard-Garris et al. [64]	*N* = 12,379 49.6% female Age range = 24–32 years	Juvenile detention facilities and correctional facilities, CG: general population sample	< 18 years	N/R	United States	Retrospective	N/A	Survey	Lifetime	Anxiety disorders, depressive disorders, post-traumatic stress disorders
Ramchand et al. [72]	*N* = 395 13.7% female Age range = 20–24 years	Juvenile detention facilities	N/R	Range = 9–12 months	United States	Prospective	12	DSM-IV	1 year	Depressive disorders, conduct disorders, attention-deficit hyperactivity disorder substance-use disorders
Verbruggen et al. [73]	*N* = 251 Mean age = 34.9 years	Correctional facilities	Mean age = 15 years (SD = 1.6)	Mean = 17 months (SD = 11.8)	Europe	Prospective	49.7	CES-D, CIDI	1 year	Depressive disorders, substance-use disorders, post-traumatic stress disorders, psychotic disorders

*DISC-IV* Diagnostic Interview Schedule for Children Version IV, *DIS-IV* Diagnostic Interview Schedule for DSM-IV, *WMH-CIDI* The World Mental Health Organization Composite International Diagnostic Interview, *GHQ* General Health Questionnaire, *ICD* International Classification of Diseases, *CIDI-SF* Composite International Diagnostic Interview-Short Form, *CAGE* Scale Cut-Annoyed-Guilty-Eye Scale, *EPDS* Edinburgh Postnatal Depression Scale, *CCEI* Crown-Crisp Experiential Index, *CES-D* Center for Epidemiological Studies Scale for Depression, *DSM-IV* *Diagnostic and Statistical Manual of Mental Disorders* (4th ed.). *CG* control group, *N/A* not available, *N/R* not reported, *SD* standard deviation

^a^Combined study sample sizes, including all study participants, are reported

^b^Only outcome measures that are included in the meta-analysis are reported

Most studies were prospective and examined mental disorders among adults with a child welfare out-of-home care placement history (Table 1). Four studies were retrospective, and four studies examined mental disorders among adults with a juvenile justice residential care history. Seven studies investigated samples with foster care histories, one study included an additional sample with a residential child welfare care history, and nine studies included mixed samples of foster and residential child welfare care histories. Regarding exposure to child welfare out-of-home care, six studies reported that entry into care was in childhood (age ranged from 2 to 12 years across these studies), three studies reported that entry into care was in adolescence (age ranged from 13 to 17 years), and six studies did not provide information on age at entry into child welfare care. For juvenile justice out-of-home care, three studies reported that entry into care ranged from childhood to adolescence (age ranged from 10 to 17 years across these studies); only one study did not provide any information on age at entry into juvenile justice out-of-home care. The average time in child welfare out-of-home care ranged from 1 month to 15 years across six studies; nine studies did not provide any information on duration of care. The average duration of care in juvenile justice out-of-home care ranged from 9 to 17 months across two of our included studies; two studies did not provide any information on the duration of juvenile justice care. Lastly, dropout rates ranging from 1.95 to 42.3% were reported across ten studies on child welfare placement histories; the other five studies on child welfare placement histories, on the other hand, did not report dropout rates. The dropout rates in studies on juvenile justice placement histories ranged from 12 to 49.7% across three of the included studies; only one study did not provide any information on the dropout rate of the study sample.

For three studies, which reported gender-specific prevalence rates only [61–63], we calculated the combined prevalence across these subsamples in our meta-analyses [56]. We included two studies that reported only lifetime prevalence rates [48, 64], because we were able to control for childhood prevalences by conducting subgroup analyses. Finally, eight studies reported prevalence rates for the general population as a control group in addition to the prevalences in the exposed group of adults with an out-of-home placement history [61, 62, 64–69].

### Quality of studies included

The total quality scores for eligible studies ranged from 3 (*k* = 1) to 8 (*k* = 5) (see Table 2). Eleven studies were of moderate quality, seven studies of high quality, and only one study of low quality. All studies met the “Was Follow-Up Long Enough for Outcomes to Occur” quality criterion in the outcome dimension, whereas only three studies met the “Demonstration that Outcome of Interest Was Not Present at Start of Study” in the selection dimension.

**Table 2:** Quality assessment of studies included (*k* = 19) with the Newcastle–Ottawa Quality Scale

Study	Selection				Comparability	Outcome			Total score
	Representativeness of the exposed cohort	Selection of the non-exposed cohort	Ascertainment of exposure	Demonstration that outcome of interest was not present at start of study	On the basis of the design or analysis	Assessment of outcome	Was follow-up long enough for outcomes to occur	Adequacy of follow-up of cohorts	
Abram et al. [71]	1	N/A	1	0	1	1	1	1	6
Benedict et al. [70]	1	N/A	1	0	1	0	1	1	5
Björkenstam et al. [65]	1	1	1	0	2	1	1	1	8
Brown et al. [76]	1	N/A	1	0	1	1	1	1	6
Casanueva et al. [77]	1	N/A	1	1	2	1	1	1	8
Côté et al. [67]	1	1	1	0	2	1	1	1	8
Dregan and Gulliford [66]	1	1	1	0	2	0	1	0	6
Fernandez et al. [78]	1	N/A	1	0	0	0	1	0	3
Forsman et al. [47]	1	N/A	1	0	2	1	1	1	7
Heard-Garris et al. [64]	1	1	1	1	2	0	1	1	8
Hjern et al. [103]	1	N/A	1	0	1	1	1	1	6
Landers et al. [48]	1	N/A	1	0	2	0	1	1	6
Räikkönen et al. [68]	1	1	1	0	2	1	1	1	8
Ramchand et al. [72]	1	N/A	1	0	1	1	1	1	6
Southerland et al. [69]	1	1	1	0	2	1	1	0	7
Teyhan et al. [61]	0	1	0	1	2	0	1	1	6
Verbruggen et al. [73]	1	N/A	1	0	2	1	1	0	6
Vinera and Taylor [62]	1	1	1	0	2	0	1	0	6
Vinnerljung and Sallnäs [63]	1	N/A	1	0	1	1	1	1	6
Total score	18	8	18	3	30	12	19	14	

*N/A* not applicable due to lack of a control group. The quality ratings are based on the standard ‘stars system’ of the Newcastle–Ottawa Quality Assessment Scale but are presented as numbers here

### Prevalence rates of mental disorders

#### Child welfare placement history

The pooled prevalence rate for any mental disorder in adults with a foster or residential care history based on child welfare placement was 29.89 [95% CI (23.36, 37.36); see Table 3]. The pooled prevalence rates for the specific disorder groups ranged from 3.32% to 17.16% across all studies, with psychotic disorders showing the lowest and depressive disorders the highest rates. Moderate to high between-study heterogeneity was found for the specific disorder groups (all *p* < 0.05; Table 3). A visual overview of the prevalence rates found in individual studies is provided in the forest plot in Supplementary Fig. 1.

**Table 3:** Random-effects model meta-analyses for prevalence rates among adults formerly in out-of-home care

Stratified by disorder	Prevalence rates (95% CI)			Heterogeneity tests			
	Number of studies	Prevalence rates	95% CI	*I*^2^	*Q*	*τ*^2^	*p* value
Any mental disorder							
Child welfare (all studies)	13	29.89	23.36, 37.36	97.70	339.98	36.15	< .001
Child welfare (excluding outlier studies)	8	31.26	26.86, 36.03	90.70	74.65	7.82	< .001
Juvenile justice (all studies)	1	44.87	42.38, 47.38	N/A	N/A	N/A	N/A
Depressive disorders							
Child welfare (all studies)	10	17.16	11.35, 25.11	97.20	339.66	56.04	< .001
Child welfare (excluding outlier studies)	8	13.97	9.93, 19.31	94.40	121.14	26.88	< .001
Juvenile justice (all studies)	3	24.36	20.29, 28.95	64.20	8.93	0.03	< .01
Substance-use disorders							
Child welfare (all studies)	12	10.39	9.00, 11.97	55.30	27.95	0.04	< .003
Child welfare (excluding outlier studies)	10	10.42	9.35, 11.61	0.00	9.05	0.00	.43
Juvenile justice (all studies)	3	49.68	28.63, 70.84	98.40	125.51	0.62	< .001
Anxiety disorders							
Child welfare (all studies)	3	10.77	4.10., 25.42	97.00	110.96	0.82	< .001
Juvenile justice (all studies)	1	13.01	10.31, 16.28	N/A	N/A	N/A	N/A
Post-traumatic stress disorders							
Child welfare (all studies)	1	10.96	8.71, 13.72	N/A	N/A	N/A	N/A
Juvenile justice (all studies)	2	5.79	4.32, 7.71	0.00	0.26	0.00	.61
Psychotic disorders							
Child welfare (all studies)	1	3.32	2.57, 4.27	N/A	N/A	N/A	N/A
Juvenile justice (all studies)	1	6.37	3.94, 10.15	N/A	N/A	N/A	N/A
Eating disorders							
Child welfare (all studies)	1	10.00	6.74, 14.60	N/A	N/A	N/A	N/A
Attention-deficit hyperactivity disorder							
Juvenile justice (all studies)	1	47.34	42.46, 52.28	N/A	N/A	N/A	N/A
Conduct disorders							
Juvenile justice (all studies)	1^a^	65.82	61.00, 70.34	N/A	N/A	N/A	N/A

*CI *confidence interval, *N/A* not applicable

^a^Rates for conduct disorders among adults with a juvenile justice history were recorded if 3 or more of 15 symptoms of the *Diagnostic and Statistical Manual of Mental Disorders* (4th ed.), were present

Our sensitivity analyses for exploring potential sources of between-study heterogeneity revealed that excluding five outlier studies [48, 63, 68–70] in the any mental disorder group and two outlier studies [48, 66] in the depressive disorders group did not change the prevalence rates or the heterogeneities substantially (Table 3). Excluding two outlier studies [62, 68] in the substance-use disorders group did increase the prevalence rate slightly, from 10.39% to 10.42%, and revealed a low and non-significant heterogeneity of 0.00%.

The asymmetry in the funnel plots for any mental disorder, depressive disorders, substance-use disorders, and anxiety disorders indicates a possible risk of overestimating the prevalence rates in this meta-analysis (see Supplementary Fig. 2). The information depicted in the funnel plots indicates that studies with lower prevalence rates might be missing from the pool of studies included. Such a pattern is typically explained by the presence of publication bias, meaning that studies with smaller and possibly non-significant prevalence rates are more likely to remain unpublished compared to studies with larger and significant findings. Our results, which summarize only the publicly available evidence, might suggest a larger prevalence rate than would be the case if all evidence, including unpublished findings, had been analyzed. However, this bias should be interpreted carefully due the small number of studies in our analysis [55].

Table 4 provides information on the subgroup analyses for the child welfare history sample. Subgroup analyses for any mental disorder showed higher prevalence rates in studies with a kinship care setting than in studies with other child welfare care settings (*p* = .02); in studies using the GHQ as an outcome measure than in studies using other measures (*p* < .001); in studies of present prevalence diagnoses than in studies with other time periods of prevalence (*p* = 0.03); and in studies of the United States than in studies of other locations (*p* = .03). Subgroup analyses for depressive disorders indicated higher prevalence rates in studies using a survey as an outcome measure than in studies using other measures (*p* < .001); in studies of lifetime prevalence diagnoses than in studies with other time periods of prevalence (*p* < .001); in retrospective studies than in prospective studies (*p* < .001); and in studies of the United States than in studies of Europe (*p* < .001).

**Table 4:** Subgroup analyses of prevalence rates stratified by disorder among adults with residential or foster care placement histories

Moderator	Number of studies	Prevalence rates	95% CI	*Q* value	*I*^2^	*p* value
Any mental disorder						
Setting				9.69		.02
Out-of-home care	5	30.91	21.94, 41.59		95.60	
Foster care	6	27.54	17.81, 40.00		98.60	
Kinship care	1	45.35	35.18, 55.92		N/A	
Adoption	1	22.63	20.51, 33.79		N/A	
Outcome measure				66.63		< .001
GHQ	2	51.39	44.38, 58.34		1.50	
The Malaise Inventory	1	24.78	20.50, 29.63		N/A	
ICD	3	19.85	12.32, 30.38		96.60	
CIDI	2	37.08	21.83, 55.44		92.70	
Hospital records	4	31.59	25.76, 38.07		90.90	
Survey	1	16.88	12.58, 22.28		N/A	
Time period of prevalence				11.38		.003
Present	11	31.70	24.26, 40.21		97.90	
1 year	1	26.08	22.73, 29.74		N/A	
Lifetime	1	16.88	12.58, 22.28		N/A	
Design				0.43		.51
Prospective	11	30.79	23.48, 39.22		97.80	
Retrospective	2	25.44	14.52, 40.68		92.60	
Location				7.34		.03
United States	5	37.18	24.33, 52.14		94.70	
Europe	7	24.67	18.74, 31.73		96.80	
Oceania	1	35.58	32.04, 39.28		N/A	
Depressive disorders						
Setting				7.60		.18
Foster care	4	17.39	6.62, 38.46		98.50	
Residential care	1	22.22	16.61, 29.06		N/A	
Foster and residential care	1	23.64	14.25, 36.57		N/A	
Out-of-home care	2	16.30	11.94, 21.84		71.10	
Adopted	1	14.96	11.21, 19.69		N/A	
Unknown	1	10.17	4.64, 20.84		N/A	
Outcome measure				153.59		< .001
ICD	2	8.60	4.20, 16.82		97.80	
The Malaise Inventory	4	19.72	16.23, 23.75		0.00	
CIDI	1	12.29	9.90, 15.16		N/A	
Edinburgh Postnatal Depression Scale	2	17.63	13.67, 22.43		33.20	
Survey	1	53.07	46.58, 59.46		N/A	
Time period of prevalence				143.69		< .001
Present	8	14.81	10.45, 20.56		93.80	
1 year	1	12.29	9.90, 15.16		N/A	
Lifetime	1	53.07	46.58, 59.46		N/A	
Design				71.92		< .001
Prospective	9	14.46	10.60, 19.40		93.70	
Retrospective	1	53.07	46.58, 59.46		N/A	
Location				71.92		< .001
United States	1	53.07	46.58, 59.46		N/A	
Europe	9	14.46	10.60, 19.40		93.70	
Substance-use disorders						
Setting				6.93		.23
Out-of-home placement	3	12.14	9.61, 15.24		62.90	
Foster care	5	9.78	8.15, 11.69		38.80	
Residential care	1	11.11	7.20, 16.76		N/A	
Foster and residential care	1	10.91	4.98, 22.23		N/A	
Adopted	1	5.33	2.79, 9.92		N/A	
Unknown type	1	8.47	3.57, 18.80		N/A	
Outcome measure				4.78		.31
CAGE Scale	5	13.04	10.36, 16.29		0.50	
ICD	2	9.82	7.14, 13.36		74.50	
CIDI	2	10.19	8.71, 11.88		0.00	
Survey	1	9.21	6.08, 13.71		N/A	
Hospital records	2	8.35	4.27, 14.35		57.40	
Time period of prevalence				0.04		.84
Present	10	10.43	8.68, 12.50		58.70	
1 year	2	10.19	8.71, 11.88		0.00	
Design				0.35		.55
Prospective	11	10.50	9.01, 12.21		58.50	
Retrospective	1	9.21	6.08, 13.71		N/A	
Location				0.17		.68
Europe	9	10.58	8.66, 12.88		61.80	
United States	3	10.05	8.68, 11.60		0.00	

.31*N/A* not applicable, *GHQ* General Health Questionnaire, *ICD* International Classification of Diseases, *CIDI* Composite International Diagnostic Interview, *CAGE scale* Cut-Annoyed-Guilty-Eye Scale

#### Juvenile justice placement history

The study of Abram et al. [71] reported a prevalence rate of 44.87 [95% CI (42.38, 47.38)] for any mental disorder in adults with a juvenile justice placement history (see Table 3). Pooled prevalence rates for the different mental disorders varied between 6% and 66%, whereas the rate of conduct disorders was the highest and the rates of both post-traumatic stress disorders and psychotic disorders were the lowest. Moderate heterogeneity was found for three studies of depressive disorders, and high heterogeneity was found for three studies of substance-use disorders. A visual overview of the prevalence rates found in individual studies is provided in the forest plot in Supplementary Fig. 3.

Reviewing individual studies that examined depressive disorders for potential sources of heterogeneity, Heard-Garris et al. [64] reported lower prevalence rates compared with the studies of Ramchand et al. [72] and Verbruggen et al. [73]. Heard-Garris et al. [64] used a retrospective study design, in contrast to the other, prospective studies; assessed lifetime rather than 1-year prevalence rates; and used a survey rather than other measures (see Table 1). Comparing the individual studies of substance-use disorders for sources of heterogeneity, Abram et al. [71] and Ramchand et al. [72] reported higher prevalence rates compared with the Verbruggen et al. [73] study. Both Abram et al. [71] and Ramchand et al. [72] examined samples from detention center settings, rather than a sample from a judicial treatment institution, and were conducted in the United States rather than Europe.

There was an indication of asymmetry in the funnel plot for depressive disorders and substance-use disorders, pointing to a possible risk of overestimating the prevalence rates in our meta-analysis due to the assumed presence of unpublished studies (i.e., publication bias). The small number of studies limits the interpretability of these results (see Supplementary Fig. 4).

### Odds for mental disorders

#### Child welfare placement history

Adults with a foster or residential care history resulting from child welfare placement showed significantly higher rates of any mental disorder [OR = 1.56, 95% CI (1.14, 2.13), *p* = .0061] compared with the general population (see Table 5). For the specific disorders, prevalence rates for depressive disorders [OR = 1.98, 95% CI (1.28, 2.89), *p* = .002], substance-use disorders [OR = 1.33, 95% CI (1.16, 1.91), *p* < .001], and anxiety disorders [OR = 1.75; 95% CI (1.20, 2.56), *p* = .004] were higher among adults with a foster or residential care history in a child welfare context than in the general population. Low to high heterogeneity was found for the various disorder groups. A visual overview of ORs found for individual studies is provided in the forest plot in Supplementary Fig. 5.

**Table 5:** Random-effects model meta-analyses for the odds of mental disorders among adults formerly in out-of-home care

Stratified by disorder	OR (95% CI)				Heterogeneity tests			
	Number of studies	OR	95% CI	*p* value	*I*^2^	*Q* value	*τ*^2^	*p* value
Any mental disorder								
Child welfare (all studies)	4	1.56	1.14, 2.13	.006	82.00	16.66	0.08	< .001
Depressive disorders								
Child welfare (all studies)	8	1.92	1.28, 2.89	.002	93.60	110.18	0.30	< .001
Child welfare (excluding outlier studies)	7	1.57	1.36, 1.80	< .001	0.00	22.48	0.00	< .001
Juvenile justice (all studies)	1	1.54	1.23, 1.91	< .001	N/A	N/A	N/A	N/A
Substance-use disorders								
Child welfare (all studies)	7	1.33	1.16, 1.52	< .001	0.00	5.19	0.00	.52
Anxiety disorders								
Child welfare (all studies)	3	1.75	1.20, 2.56	.004	75.70	8.23	0.09	.02
Juvenile justice (all studies)	1	1.07	0.81, 1.40	.64	N/A	N/A	N/A	N/A
Post-traumatic stress disorders								
Juvenile justice (all studies)	1	2.76	1.87, 4.09	< .001	N/A	N/A	N/A	N/A

*OR* odds ratio, *CI* confidence interval, *N/A* not applicable

To minimize heterogeneity between the prevalence rates of included studies, one outlier study [65] in the depressive disorders group was excluded; this did not change the OR substantially but reduced the heterogeneity (from 93.60% to 0.00%; Table 5). There was an indication of asymmetry in the funnel plot for any mental disorder, depressive disorders, and anxiety disorders, pointing to a possible risk of overestimating the prevalence rates in this meta-analysis due to unpublished studies (i.e., publication bias; see Supplementary Fig. 6).

#### Juvenile justice placement history

Only the study of Heard-Garris et al. [64] compared prevalence rates of mental disorders in adults with a juvenile justice residential care history and the general population as a control group (Table 5). Adults with a juvenile justice residential care history reported significantly higher rates of depressive disorders [OR = 1.54; 95% CI (1.23, 1.91), *p* < .001] and post-traumatic stress disorders [OR = 2.76; 95% CI (1.87, 4.09), *p* < .001] than the general population (Table 5).

## Discussion

The aim of our meta-analytic review was to summarize the available evidence on the occurrence of mental disorders among adults previously in out-of-home care, and, where possible, to compare these rates with the occurrence of mental disorders in control groups.

Our meta-analysis revealed a pooled prevalence rate of 30% for any mental disorder in adults with a foster or residential child welfare placement history. This figure is consistent with the findings of previous studies regarding mental health in young adults leaving out-of-home care [6, 7, 41]. However, the rate is highly elevated compared to the previously calculated prevalence rate of 18% for any mental disorder in meta-analyses examining the general adult population [27, 74]. This observation reflects the fact that our results are based on studies that used a general population control group in addition to the exposed group, and which showed an increased risk of the occurrence of mental disorders in adults with a history of out-of-home placement compared to adults in the general population.

Our analyses also showed that the prevalence rate for any mental disorder varied largely across individual studies, with high heterogeneity in our random-effects model. This may be due to the fact that the studies subsumed various specific mental disorders under one general mental disorder measure, mixing together disorders that are known to have different general prevalences in and of themselves. Nonetheless, the pooled prevalence rate of 30% for any mental disorder among adults with a child welfare placement history is lower than the rate of 49% for any mental disorder reported in a previous meta-analysis examining mental disorders during child welfare system involvement [3].

Our stratified analyses, which examined pooled prevalence rates of specific disorders for adults with a child welfare placement history, showed that these rates varied largely, between 3% for psychotic disorders and 17% for depressive disorders. The distribution of the rates across individual disorders is similar to the distribution found in previous meta-analyses and primary studies examining mental disorders within samples from child welfare out-of-home care [3, 21, 75]. Furthermore, studies examining various mental disorders among adults with a child welfare placement history have shown almost a twofold increase in the risk of depressive disorders, anxiety disorders, and substance-use disorders compared with the general population.

Finally, our meta-analysis showed a pooled prevalence rate of 45% for any mental disorder in adults with a juvenile justice placement history. The pooled prevalence for specific disorders in this sample again varied largely, between 6% for post-traumatic stress disorders and 66% for conduct disorders. These rates are within the range of prevalence rates for mental disorders obtained in previous prospective studies of adults with a juvenile justice placement history [35, 38]. Our random-effects models revealed moderate heterogeneity for depressive disorders and high heterogeneity for substance-use disorders, which is in line with the heterogeneity reported in a previous meta-analysis of mental disorders among adolescents in correctional facilities [4]. We were not able to conduct subgroup analyses for this sample because we were only able to include four studies examining mental disorders among adults with a juvenile justice placement history in our study [64, 71–73].

Our meta-analyses revealed that adults formerly in out-of-home care are overall more likely to show mental disorders compared with the general adult population, regardless of setting. However, results varied across individual studies and indicated low to high heterogeneity between effect size estimates, mainly due to methodological differences between studies (i.e., setting of care, time period of prevalence, and study design). Due to various moderator variables, our meta-analysis found comparable levels of heterogeneity to those in previous meta-analyses of both child welfare and juvenile justice samples [3, 4].

The elevated rates of mental disorders among adults previously in out-of-home care may be explained by the use of samples in the stage of young adulthood (18–25 years) in eight studies [63, 65, 67, 69, 70, 72, 76, 77], compared with the highest mean age of 72 years in the study sample of Fernandez et al. [78]. For example, there was a variation in the age distribution (18–72 years) of samples between individual studies: as young adults may face various challenges at transition to adulthood, they might be at higher risk of mental disorders compared with older adults [30], which might mean that our estimated prevalence rates are higher than is actually the case. Although mental health services can offer protective benefits, the use of such services tends to decline as young people leave care [7]. Presumably, young adults leaving care appreciate their freedom and may be reluctant to participate in any kind of foster or residential care—including mental health services—anymore [79]. On the other hand, however, the transition to adulthood might make it difficult for young adults to navigate new healthcare systems where they have to look after themselves after leaving care all of a sudden. However, more studies with greater variation in the age of previously out-of-home placed samples are needed to examine the effect of age on the prevalence estimates.

One of the main issues in research on the effects of child welfare and juvenile justice care involvement to date is the lack of effective control groups with comparable risk factors. A comparison of long-term trajectories between samples with a child welfare or juvenile justice placement history and normative samples should be interpreted cautiously, since samples with out-of-home placement histories are populations disadvantaged and marginalized by a disproportionate concentration of cumulative childhood risk factors for various mental disorders. Our meta-analysis has shown that the odds for mental disorders among adults with an out-of-home care history are lower than in the meta-analysis of Hughes et al. [80], which revealed ORs ranging from 3.70 for anxiety to 10.22 for problematic drug use in adults with a high burden of cumulative childhood adversities. In addition, the lower rates of mental disorders in adulthood than those found in previous reviews of samples from these groups [3, 4] might be explained by the fact that the transition period into adulthood is accompanied by strengthening and protective factors originating in the child welfare or juvenile justice care systems. To make conclusive statements about the odds of mental disorders among child welfare and juvenile justice samples, future studies, including samples with levels of psychosocial burden comparable to those of adults with out-of-home care histories, are needed.

### Implications

As previous studies have shown that various care settings (including foster or residential care) can be effective for the healthy development of adolescents with an out-of-home care history [24, 26], our findings add to these results by indicating that there is a need for more studies examining protective factors that contribute to a positive development of mental health into adulthood among individuals with a history in various care settings. Furthermore, more research is needed to compare the effectiveness for adult mental health of out-of-home care with the mental health of adults who were highly burdened in childhood but were not placed out-of-home as effective control groups. Nonetheless, children and adolescents taken out-of-home show psychosocial burdens and experiences different from those who remain in their parental homes. The fact that 30% of adults with a history of out-of-home care in the child welfare system show any mental disorder is an important reminder of the need to enhance foster and residential care in childhood. The eligible studies on mental disorders in adults with a juvenile justice placement history were considerably lower in number than the studies on adults with a child welfare placement history, and generally not of a high quality. We, therefore, have less confidence in our findings in this respect, and more research is needed regarding adults with histories of juvenile justice placement. Furthermore, previous studies have indicated that crossover youths show higher levels of negative trajectories in care and greater exposure to psychosocial burden [15, 17], but we were not able to obtain information on dual involvement in care systems. Our findings thus indicate that more studies with standardized study designs and sample definitions are needed to estimate the occurrence of mental disorders across care systems to identify protective and risk factors for individuals with out-of-home care histories.

### Limitations

The results of our meta-analysis have some limitations. First, the small number of studies for some mental disorders limits the interpretation of our effect sizes. We could not examine the effect of continuous moderators in a meta-regression analysis (i.e., percentage of females, mean age of sample, age at entry into care, duration of care, and dropout rates), because conducting meta-regression analysis with *k* < 10 studies is not considered valid, and therefore, not recommended [55]. In addition, it is not recommended to conduct meta-regressions with person-level (as opposed to study-level) variables in meta-analyses (e.g., age as a continuous predictor patient-level variable; [81]). A meta-regression based on the study means of patient-level variables is considered problematic, because these findings are prone to bias; this is often described as the ecological fallacy [81]. To come to valid conclusions in meta-analyses using individual-based predictors (like age), individual patient data would be needed, to conduct individual patient data meta-analyses. This, however, was not the goal of the present meta-analytic investigation. Furthermore, between 7 and 11 of the studies we included did not report information on exposure to out-of-home care, such as mean age at entry into care, duration of care, and dropout rates. However, previous studies have shown that early age at entry and a longer duration of care are associated with better mental health outcomes in youth care [82, 83]. Only one study reported the number of placements [66], and none of the included studies provided information on the disruption or breakdown of care, both of which have been associated with mental health outcomes [84–86]. Thus, future studies should provide more information about relevant characteristics of out-of-home care to identify their effects on subsequent mental health. In addition, we could not discern any influence of other relevant personal moderator variables (e.g., adverse childhood experiences) associated with the effect sizes [4, 21, 49, 87].

Second, our meta-analyses showed low to high heterogeneity for the individual disorders, indicating variation in study outcomes due to methodological differences between studies. In 15 of the studies we included, the diagnoses were based on diagnostic interviews, and four of the studies used screening scales instead [61, 66, 70, 73]. Although screening scales could be considered less valid than clinically defined diagnoses, previous validation studies have shown that the screening scales used in the studies we included were valid when compared with clinically derived diagnoses [88–93]. For some disorders, some individual studies contributed more to between-study heterogeneity than others; this was reduced if these outlier studies were excluded.

Third, the small number of studies in our funnel plots further indicated possible publication bias, suggesting that studies with smaller and possibly non-significant prevalence rates are more likely to remain unpublished compared to studies with larger and significant findings. If this is so, our results summarizing only the publicly available evidence might suggest a larger prevalence rate than would be the case if all the evidence, including unpublished findings, had been analyzed.

Fourth, we were not able to compare the prevalence rates of mental disorders among adults with a foster care history with those among adults with a residential child welfare placement history, because only one of the studies included a sample with residential child welfare placement history in addition to a sample with foster care history [66]. Future studies might, therefore, distinguish between foster and group care samples to directly compare mental disorders between the two groups. Likewise, none of the studies we included reported information on the quality of structures or processes of care, which have been shown to explain a great part of the variance in outcomes across care systems [8, 94, 95].

Fifth, the extent to which a child welfare or juvenile justice authority is involved in out-of-home care depends on the policy and legislation of the country in question. The differing organization of child welfare and juvenile justice systems between countries complicates the comparison and pooling of results for samples from different jurisdictions, and thus should be interpreted with caution.

Sixth, our findings do not allow conclusions to be drawn regarding trajectories or treatment effects, as only three studies [64, 76, 77] assessed mental disorders during and after out-of-home care.

Finally, since only one study [67] examined the comorbidity of mental disorders, we were not able to include this relevant factor in our analyses [96, 97]. For example, Dölitzsch et al. [20] showed that 74% of children and adolescents in residential care had a psychiatric disorder, and that 60% of those individuals also fulfilled the criteria for more than one diagnosis. In addition, higher risks for comorbid disorders in adulthood have been reported in other studies [27, 98]. Therefore, future research should investigate the comorbidity of disorders after leaving care to improve etiological models and interventions to account for this complexity.

## Conclusions

To our knowledge, this is the first comprehensive review and meta-analysis summarizing adult mental disorders in the high-risk group of individuals with a child welfare or juvenile justice out-of-home care history. The main finding is that one-third of adults with a child welfare foster or residential care history show any mental disorder. In comparison, almost half of adults with a juvenile justice residential care history fulfill the criteria for a mental disorder. Both those rates are significantly higher than the rates found in studies of the general adult population. However, our findings suggest that the level of any mental disorder in adulthood for individuals in out-of-home care might be lower than the level of mental disorders found in previous meta-analyses of out-of-home care [3, 4] or in psychosocially highly burdened adults without a history of out-of-home care [80].

The child and adolescent psychiatry and out-of-home care systems are tasked with supporting young people’s mental health by providing a successful transition into young adulthood, which is accompanied by various difficulties during this vulnerable period [29]. The scarcity of evidence regarding specific disorders in out-of-home placed populations highlights the need for more longitudinal research that estimates the prevalence rates of mental disorders with standardized assessments and with standardized definitions and quality characteristics of care, thereby reaching conclusive findings about the trajectories of the specific disorders. Future studies on the development of mental disorders in care systems may help to identify protective factors among individuals with mental disorders, so as to further support a successful transition to an independent adult life. For instance, future studies should investigate the effect of close cooperation between child and adolescent psychiatric services and care systems in offering a combination of environment-orientated and evidence-based treatments for individuals leaving care (i.e., liaison services; [24, 99–102]). Such results would be important for shaping interventions that support a successful transition to an independent adult life. Due to the great differences in child protection and juvenile justice law and policies between countries and jurisdictions, international studies on the trajectories of mental disorders after leaving care are challenging but, nonetheless, badly needed.

## Supplementary Information

Below is the link to the electronic supplementary material.

Supplementary file1 (DOCX 869 KB)

## Data Availability

Not applicable.
