# Supplementary material for: Mental disorders among adults formerly in out-of-home care: a systematic review and meta-analysis of longitudinal studies
Source: Eur Child Adolesc Psychiatry. 2021 Jun 24;31(12):1963–82. doi: 10.1007/s00787-021-01828-0 (PMC9663399; doi:10.1007/s00787-021-01828-0)
Supplement: Supplementary file 1 — Supplementary file1 (DOCX 869 KB) [file 787_2021_1828_MOESM1_ESM.docx]

# Supplementary Material

**Mental Disorders Among Adults Formerly in out-of-Home Care: A Systematic Review and Meta-Analysis of Longitudinal Studies**

*Journal:* European Child & Adolescent Psychiatry

*Authors:* Süheyla Seker, Cyril Boonmann, Heike Gerger, Lena Jäggi, Delfine d’Huart, Klaus Schmeck, and Marc Schmid

*Corresponding author:* Süheyla Seker; Department of Child and Adolescent Psychiatry Research, Psychiatric University Hospitals, University of Basel, Switzerland; [sueheyla.seker@upk.ch](mailto:sueheyla.seker@upk.ch)

# Contents

| **Supplementary Content 1.** | MOOSE (Meta-analyses of Observational Studies in Epidemiology) Checklist |
| --- | --- |
| **Supplementary Content 2.** | Deviations of final review from initial PROSPERO protocol |
| **Supplementary Content 3.** | Search Strategy by Database |
| **Supplementary Figure 1.** | Forest Plot of Prevalence Rates Among Adults With Child Welfare History |
| **Supplementary Figure 2.** | Funnel Plots of Prevalence Rates Stratified by Disorder Among Adults With Child Welfare History |
| **Supplementary Figure 3.** | Forest Plot of Prevalence Rates Among Adults With Juvenile Justice History |
| **Supplementary Figure 4.** | Funnel Plots of Prevalence Rates Stratified by Disorder Among Adults With Juvenile Justice History |
| **Supplementary Figure 5.** | Forest Plots of Odds Ratios Stratified by Disorder Among Adults With Child Welfare History |
| **Supplementary Figure 6.** | Funnel Plots of Odds Ratios Stratified by Disorder Among Adults With Child Welfare History |

**Supplementary Content 1.** MOOSE (Meta-analyses of Observational Studies in Epidemiology) Checklist

| **Item No.** | **Reporting Criteria** | **Reported on Page No.** |
| --- | --- | --- |
| Reporting of background | | |
| 1 | Problem definition | 3, 5 |
| 2 | Hypothesis statement | 5 |
| 3 | Description of study outcome(s) | 3, 6 |
| 4 | Type of exposure or intervention used | 3, 5, 6 |
| 5 | Type of study designs used | 4, 5 |
| 6 | Study population | 4, 5 |
| Reporting of search strategy | | |
| 7 | Qualifications of searchers (eg, librarians and investigators) | 5 |
| 8 | Search strategy, including time period included in the synthesis and key words | 5 |
| 9 | Effort to include all available studies, including contact with authors | 6, 7 |
| 10 | Databases and registries searched | 5 |
| 11 | Search software used, name and version, including special features used (eg, explosion) | 5 |
| 12 | Use of hand searching (eg, reference lists of obtained articles) | N/A |
| 13 | List of citations located and those excluded, including justification | 8, Figure 1 |
| 14 | Method for addressing articles published in languages other than English | 5 |
| 15 | Method of handling abstracts and unpublished studies | 5, 6 |
| 16 | Description of any contact with authors | 7 |
| Reporting of methods | | |
| 17 | Description of relevance or appropriateness of studies assembled for assessing the hypothesis to be tested | 5, 6 |
| 18 | Rationale for the selection and coding of data (eg, sound clinical principles or convenience) | 6, 7 |
| 19 | Documentation of how data were classified and coded (eg, multiple raters, blinding and interrater reliability) | 6 |
| 20 | Assessment of confounding (eg, comparability of cases and controls in studies where appropriate) | 6 |
| 21 | Assessment of study quality, including blinding of quality assessors, stratification or regression on possible predictors of study results | 7 |
| 22 | Assessment of heterogeneity | 6 |
| 23 | Description of statistical methods (eg, complete description of fixed or random effects models, justification of whether the chosen models account for predictors of study results, dose-response models, or cumulative meta-analysis) in sufficient detail to be replicated | 7, 8 |
| 24 | Provision of appropriate tables and graphics | Tables 1-5, Figure 1, Supplementary Figures 1-6 |
| Reporting of results | | |
| 25 | Graphic summarizing individual study estimates and overall estimate | Supplementary Figures 1, 3, 5 |
| 26 | Table giving descriptive information for each study included | Table 1 |
| 27 | Results of sensitivity testing (eg, subgroup analysis) | 10 |
| 28 | Indication of statistical uncertainty of findings | 10, 11, 12 |

| **Item No.** | **Reporting Criteria** | **Reported on Page No.** |
| --- | --- | --- |
| Reporting of discussion | | |
| 29 | Quantitative assessment of bias (eg, publication bias) | 10, 11, 12, 16, Supplementary Figures 2,4,6 |
| 30 | Justification for exclusion (eg, exclusion of non-English language citations) | 5 |
| 31 | Assessment of quality of included studies | 7, 9, Table 2 |
| Reporting of conclusions | | |
| 32 | Consideration of alternative explanations for observed results | 12-15 |
| 33 | Generalization of the conclusions (ie, appropriate for the data presented and within the domain of the literature review) | 15-17 |
| 34 | Guidelines for future research | 14, 15, 17 |
| 35 | Disclosure of funding source | 18 |

**Supplementary Content 2.** Modifications of the initial PROSPERO protocol

During the review process, modifications were made to the planned analyses and review process, which led to some deviations from the initial protocol registered at PROSPERO. The main deviations from the initial Protocol are listed below. The Protocol on PROSPERO has been updated.

1. S**earches:** We excluded Scopus and finalized our study search on four electronic data bases (i.e., PsycINFO, EMBASE, PubMed and Web of Science).
2. **Types of studies to be included:** Due to a considerable number of studies with a retrospective design found in our study search, we decided to include retrospective and prospective longitudinal studies in our review.
3. **Measures of effect:** In addition to providing precentages of mental disorders among adults formerly in out-of-home care, we calculated odds for having a mental disorder among adults formerly in out-of-home care compared with available control groups.
4. **Data extraction (selection and coding):** According to reviewers’ input, we extracted ‘age at entry into care (in years)’ as additional information of exposure to care and included this variable in the description of the study characteristics.
5. **Strategy for data synthesis:** Most studies only reported prevalence rates of mental disorders among adults with out-of-home care history in adulthood. Therefore, percentages of mental disorders for the follow-up measurement were synthesized. Additionally, we used the subset of studies that included control groups. For the calculation of odds ratios (OR), we extracted the 2 × 2 cross-tabulation data for number of cases and non-cases for the control group as well (i.e., adults without an out-of-home care history).
6. **Analysis of subgroups or subsets:** It is not recommended to conduct meta-regressions with person-level (as opposed to study-level) variables in meta-analyses (e.g., age as a continuous predictor patient-level variable), because such analyses are prone to the ecological fallacy [1]. Therefore, we did not conduct meta-regression analyses as initially intended.
7. **Any additional information:** We adhered to the Meta-Analyses of Observational Studies in Epidemiology (MOOSE) guidelines [2] instead of the Preferred Reporting Items for Systematic Reviews and Meta-Analyses (PRISMA; [3]) statement as initially intended.

**Supplementary Content 3.** Search Strategy by Database

**PubMed**

(welfare[TIAB] or “foster care”[TIAB] or “residential care home”[TIAB] or “residential care homes”[TIAB] or “out-of-home”[TIAB] or “group home”[TIAB] or “group homes”[TIAB] or “looked-after”[TIAB] or “juvenile justice”[TIAB] or detention[TIAB])

AND

(child[TIAB] or children[TIAB] or youth[TIAB] or “Child Welfare”[MH])

AND

(mental[TIAB] or psychiatric[TIAB] or emotional[TIAB] or behavioral[TIAB])

AND

(health[TIAB] or disorder[TIAB] or disorders[TIAB] or ill[TIAB] or problem[TIAB] or problems[TIAB] or distress[TIAB] or distresses[TIAB] or “attention deficit and hyperactivity”[TIAB] or ADHD[TIAB] or “anxiety disorder”[TIAB] or “anxiety disorders”[TIAB] or anxiety[TIAB] or depression[TIAB] or depressive[TIAB] or “posttraumatic stress”[TIAB] or “post-traumatic stress”[TIAB] or PTSD[TIAB] or “substance use disorder”[TIAB or SUD[TIAB] or suicide[TIAB] or suicidal[TIAB] or criminal[TIAB] or criminals[TIAB] or “Mental Health”[MH])

AND

(“Case-Control Studies”[MH] OR “retrospective studies”[MH] OR “Control Groups”[MH] OR (case[TIAB] AND control[TIAB]) OR (cases[TIAB] AND controls[TIAB]) OR (cases[TIAB]

AND

controlled[TIAB]) OR (case[TIAB] AND comparison[TIAB]) OR (cases[TIAB] AND comparisons[TIAB]) OR “control group”[TIAB] OR “control groups”[TIAB] OR “cohort studies”[MH] OR “longitudinal studies”[MH] OR “follow-up studies”[MH] OR “prospective studies”[MH] OR “retrospective studies”[MH] OR cohort[TIAB] OR longitudinal[TIAB] OR prospective[TIAB] OR retrospective[TIAB])

NOT

(“Literature Review”[TIAB] or Review)

**PsycInfo**

((welfare or “foster care” or “residential care home*” or “out-of-home” or “group home*” or “looked-after” or “juvenile justice” or

detention).ti,ab,id.)

AND

((child* or youth).ti,ab,id or (“Child Welfare”).sh or (“Child Welfare”).mh)

AND

((mental or psychiatric or emotional or behavioral).ti,ab,id.)

AND

((health or disorder* or ill or problem* or distress* or “attention deficit and hyperactivity*” or ADHD or “anxiety disorder*” or anxiety or depress*

or “posttraumatic stress” or “post-traumatic stress” or PTSD or “substance use disorder” or SUD or uicide* or criminal*).ti,ab,id or (“Mental Health”).sh

or (“Mental Health”).mh)

AND

(((case* adj5 control*) or (case adj3 comparison*) or “case-comparison” or “control group*” or cohort or longitudinal or prospective or retrospective).ti,ab,id.

or (“longitudinal study” or “prospective study” or “retrospective study”).md.)

NOT

((“Literature Review”).md.)

**Embase**

((welfare or “foster care” or “residential care home*” or “out-of-home” or “group home*” or “looked-after” or “juvenile justice” or

detention).ti,ab,kw.)

AND

((child* or youth).ti,ab,kw or (“child welfare”).sh)

AND

((mental or psychiatric or emotional or behavioral).ti,ab,kw.)

AND

((health or disorder* or ill or problem* or distress* or “attention deficit and hyperactivity*” or ADHD or “anxiety disorder*” or anxiety or depress*

or “posttraumatic stress” or “post-traumatic stress” or PTSD or “substance use disorder” or SUD or uicide* or criminal*).ti,ab,kw or (“mental health”).sh)

AND

(((case* adj5 control*) or (case adj3 comparison*) or “case-comparison” or “control group*” or cohort or longitudinal or prospective or retrospective).ti,ab,kw.

or (“longitudinal study” or “prospective study” or “retrospective study”).sh.)

NOT

((“Literature Review”).ti,ab,kw.)

**Web of Science**

(welfare or “foster care” or “residential care home*” or “out-of-home” or “group home*” or “looked-after” or “juvenile justice” or detention)

AND

(child* or youth)

AND

(mental or psychiatric or emotional or behavioral)

AND

(health or disorder* or ill or problem* or distress* or “attention deficit and hyperactivity*” or ADHD or “anxiety disorder*” or anxiety or depress*

or “posttraumatic stress” or “post-traumatic stress” or PTSD or “substance use disorder” or SUD or uicide* or criminal*)

AND

((case* near/3 control*) or (case near/2 comparison*) or “case-comparison” or “control group*” or cohort or longitudinal or prospective or retrospective)

NOT

(“Literature Review”)

**Supplementary Figure 1.** Forest Plot of Prevalence Rates Among Adults With Child Welfare History


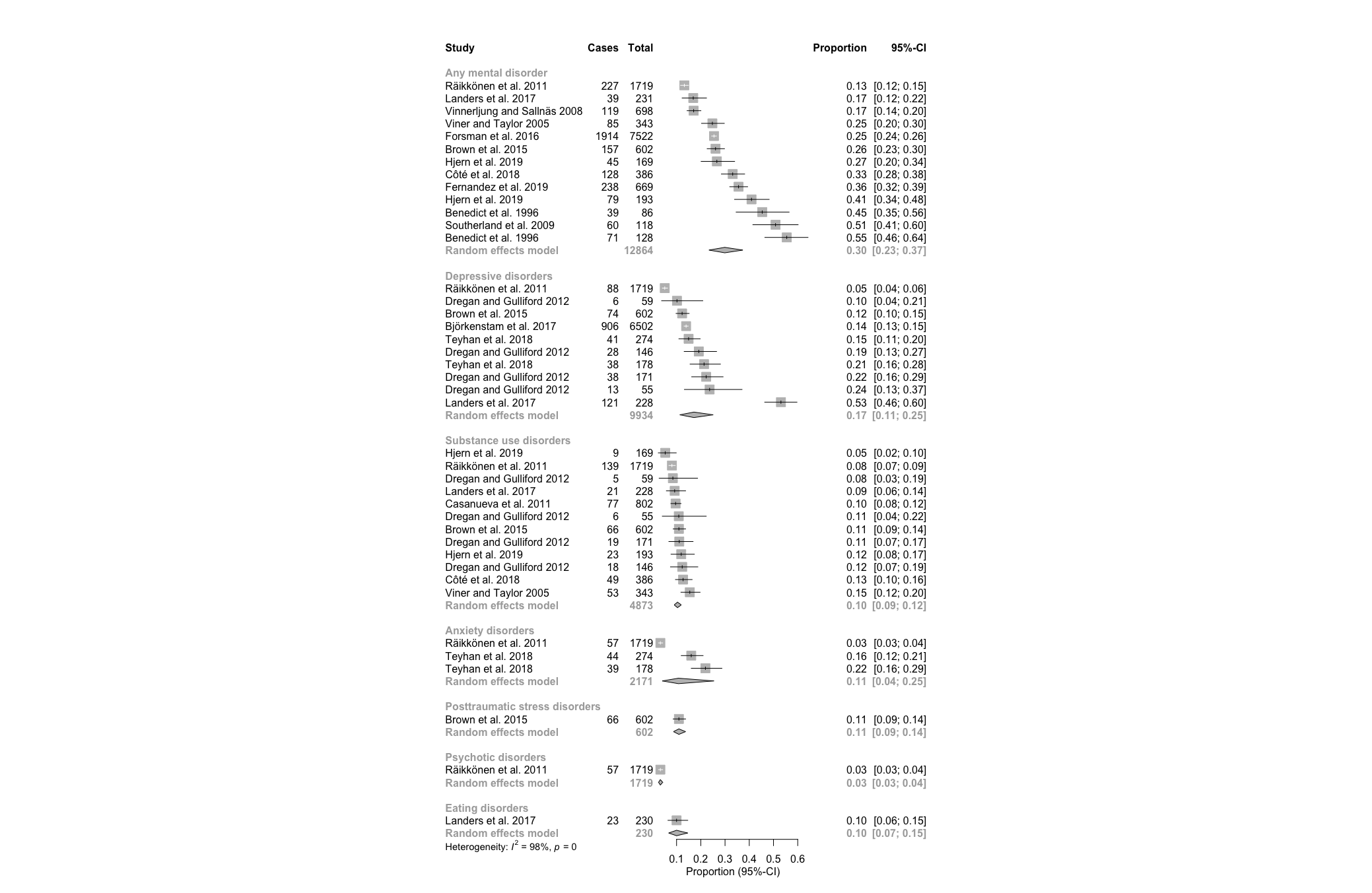


**Supplementary Figure 2.** Funnel Plots of Prevalence Rates Stratified by Disorder Among Adults With Child Welfare History. The x-axis shows logit transformed proportion and the y-axis is the standard error. The asymmetry indicates a possible publication bias towards positive findings.

**Figure 2.1** Any Mental Disorder **Figure 2.2** Depressive Disorders

**Figure 2.3** Substance-Use Disorders **Figure 2.4** Anxiety disorders

**Supplementary Figure 3.** Forest Plot of Prevalence Rates Among Adults With Juvenile Justice History

**
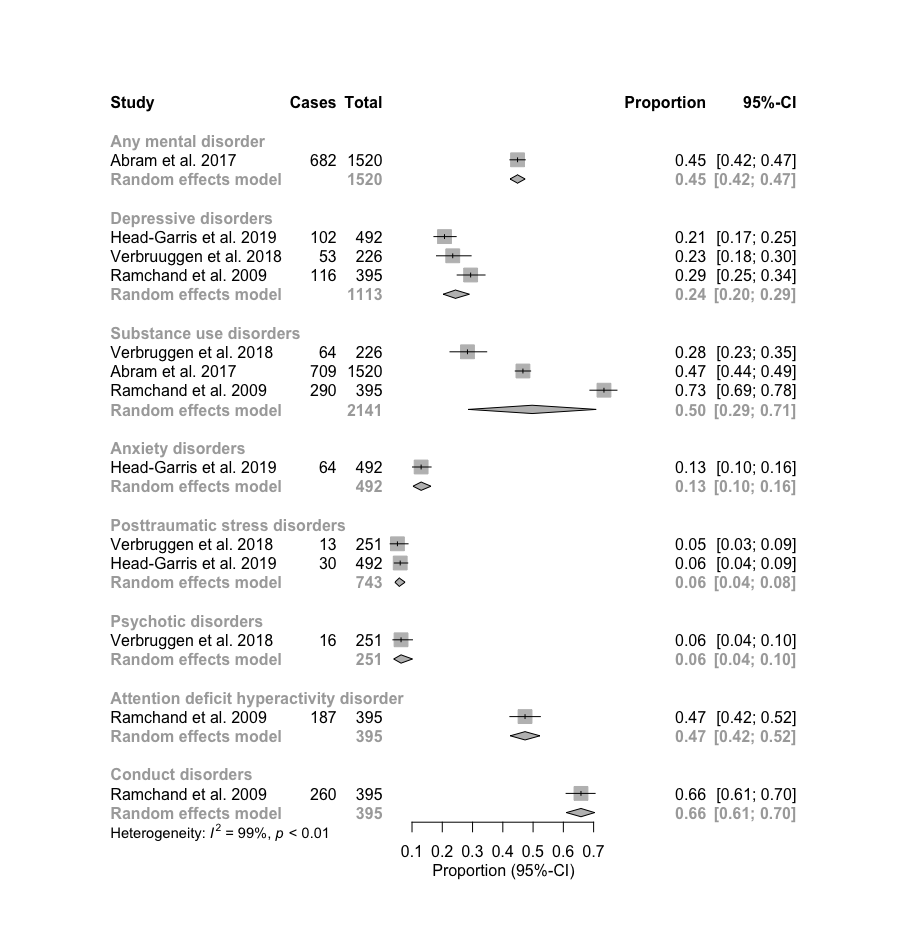
**

**Supplementary Figure 4.** Funnel Plots of Prevalence Rates Stratified by Disorder Among Adults With Juvenile Justice History. The x-axis shows logit transformed proportion and the y-axis is the standard error. The asymmetry indicates a possible publication bias towards positive findings.

**Figure 4.1** Depressive Disorders **Figure 4.2** Substance-Use Disorders

**Supplementary Figure 5.** Forest Plots of Odds Ratios Stratified by Disorder Among Adults With Child Welfare History


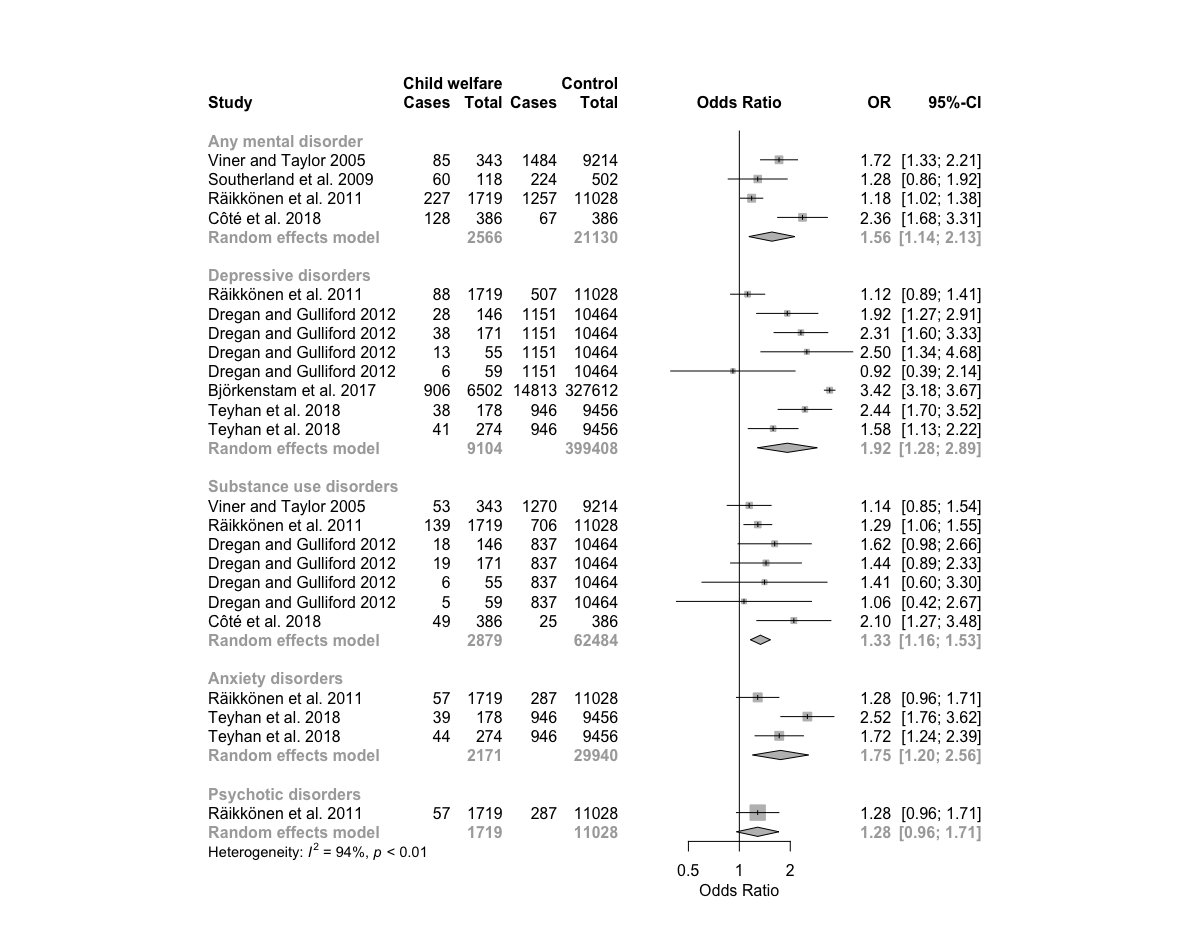


**Supplementary Figure 6.** Funnel Plots of Odds Ratios Stratified by Disorder Among Adults With Child Welfare History. The x-axis shows the odds ratio, and the y-axis is the standard error. The asymmetry indicates a possible publication bias towards positive findings.

**Figure 6.1** Any mental disorder

**Figure 6.2** Depressive Disorders

**References**

1. Thompson SG, Higgins JP (2002) How should meta-regression analyses be undertaken and interpreted? Stat Med 21:1559-1573. doi:10.1002/sim.1187

2. Stroup DF, Berlin JA, Morton SC, Olkin I, Williamson GD, Rennie D, Moher D, Becker BJ, Sipe TA, Thacker SB (2000) Meta-analysis of observational studies in epidemiology: A proposal for reporting. JAMA 283:2008-2012. doi:10.1001/jama.283.15.2008

3. Moher D, Liberati A, Tetzlaff J, Altman DG, Group P (2009) Preferred reporting items for systematic reviews and meta-analyses: the PRISMA statement. PLoS Med 6:e1000097. doi:10.1371/journal.pmed.1000097
